# Supplementary material for: Evaluating the impact of COVID-19 on routine childhood immunizations coverage in Zambia
Source: PLOS Glob Public Health. 2024 Jul 30;4(7):e0003407. doi: 10.1371/journal.pgph.0003407 (PMC11288450; doi:10.1371/journal.pgph.0003407)

**S4 Fig.** Vaccine coverage before and after COVID-19 pandemic. Data points represent monthly rates of Pentavalent dose 1, 2 & 3 between 2017 and 2022. Gray shaded area depicts the onset of COVID-19 pandemic in Zambia. Dashed lines represent fitted estimates using a linear step change model. The curved lines represents fitted values for seasonally adjusted models. Vaccine coverage before and after COVID-19 pandemic.


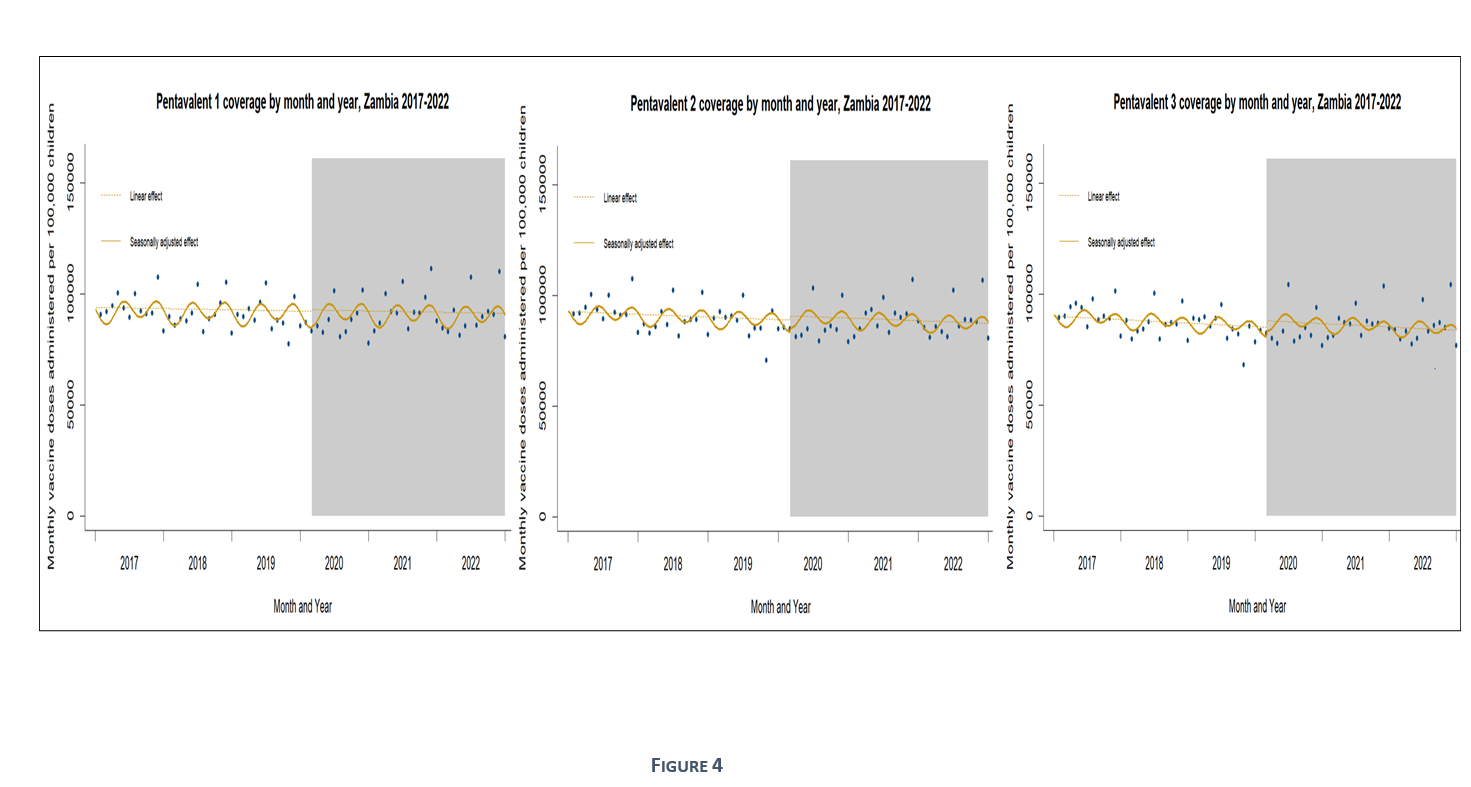

Supplement: S4 Fig — Data points represent monthly rates of Pentavalent dose 1, 2 & 3 between 2017 and 2022. Gray shaded area depicts the onset of COVID-19 pandemic in Zambia. Dashed lines represent fitted estimates using a linear step change model. The curved lines represents fitted values for seasonally adjusted models. Vaccine coverage before and after COVID-19 pandemic. (DOCX) [file pgph.0003407.s004.docx]
