# Supplementary material for: Evaluating the impact of COVID-19 on routine childhood immunizations coverage in Zambia
Source: PLOS Glob Public Health. 2024 Jul 30;4(7):e0003407. doi: 10.1371/journal.pgph.0003407 (PMC11288450; doi:10.1371/journal.pgph.0003407)
Supplement: S1 Table — (DOCX) [file pgph.0003407.s007.docx]

| Month | 2017 | 2018 | 2019 | 2020 | 2021 | Feb-22 |
| --- | --- | --- | --- | --- | --- | --- |
| January | 86.9 | 87.9 | 86.9 | 96.3 | 91.8 | 98.9 |
| February | 84.4 | 88.4 | 84.4 | 96.2 | 96.6 | 99.3 |
| March | 84.1 | 88.1 | 84.1 | 96.2 | 97.2 | 99.2 |
| April | 84.3 | 94.3 | 94.3 | 96.4 | 97.5 | 98.2 |
| May | 84.2 | 94.2 | 94.2 | 96.1 | 97.6 | 99.2 |
| June | 90.4 | 94.4 | 94.4 | 96.1 | 97.3 | 98.9 |
| July | 89.2 | 94.1 | 94.1 | 96.3 | 97.4 | 99.2 |
| August | 84.5 | 94.5 | 94.5 | 96.4 | 97.9 | 99.1 |
| September | 89.6 | 94.6 | 94.6 | 96.1 | 98.1 | 99.3 |
| October | 90.0 | 95.0 | 95.0 | 94.2 | 96.1 | 99.2 |
| November | 90.0 | 95.0 | 95.0 | 95.5 | 95.8 | 99.0 |
| December | 89.8 | 95.0 | 95.0 | 95.6 | 97.3 | 99.5 |

**S1 Table. Data Completeness from 2017 to 2022 by Month**
