# Supplementary material for: Evaluating the impact of COVID-19 on routine childhood immunizations coverage in Zambia
Source: PLOS Glob Public Health. 2024 Jul 30;4(7):e0003407. doi: 10.1371/journal.pgph.0003407 (PMC11288450; doi:10.1371/journal.pgph.0003407)
Supplement: S2 Table — (DOCX) [file pgph.0003407.s008.docx]

| Type of  Vaccine | Mean Monthly Count | | Mean Monthly Rate  per 100, 000 | | Slope Change  pre-COVID-19 | PValue^b^ | Slope Change  during-COVID-19 | PValue^b^ | Step Change | P-Value |
| --- | --- | --- | --- | --- | --- | --- | --- | --- | --- | --- |
|  | **Before**  **COVID-19** | **After**  **COVID-19** | **Before COVID-19** | **After COVID-19** | **RR (95% CI)^C^** |  | **RR(95%CI)^C^** |  | **RR (95%CI)^C^** |  |
| BCG | 55, 123 | 58, 103 | 101,108 | 100,782 | 0.997(0.994- 0.997) | 0.126 | 1.001(1.000-1.011) | 0.016 | 0.78(0.63-0.97) | 0.028 |
| Measles  dose 1 | 53, 465 | 56, 202 | 98,041 | 97,498 | 0.998(0.995-1.003) | 0.609 | 1.003(0.998-1.010) | 0.253 | 0.85(0.65-1.01) | 0.226 |
| Measles  dose 2 | 53, 465 | 39,449 | 98,041 | 68,435 | 0.998(0.995-1.003) | 0.620 | 1.00(0.997-1.010) | 0.277 | 0.59 (0.43-0.80) | 0.0001 |
| DPT 1 | 5, 088 | 58, 054 | 99,206 | 100,713 | 0.998(0.995-1.000) | 0.096 | 1.006(1.001-1.008) | 0.034 | 0.84(0.72-0.99) | 0.043 |
| DPT 2 | 52, 915 | 56, 623 | 97,057 | 98,232 | 0.997(0.995-0.998) | 0.0001 | 1.005(1.002-1.007) | 0.121 | 0.84(0.72-0.98) | 0.028 |
| DPT 3 | 51,148 | 54, 823 | 93,815 | 95,113 | 0.997(0.995-0.998) | 0.034 | 1.004(1.001-1.008) | 0.007 | 0.85(0.73-0.99) | 0.044 |
| Rota 1 | 53, 633 | 44, 010 | 98,366 | 76,716 | 0.998(0.992-1.005) | 0.578 | 0.97(0.96-0.98) | 0.0001 | 5.29(3.24 -8.62) | 0.0001 |
| Rota 2 | 52, 264 | 42, 958 | 95,858 | 74,889 | 0.998(0.992-1.004) | 0.558 | 0.97(0.96-0.98) | 0.0001 | 5.31(3.29 -8.59) | 0.0001 |
| Oral poliovirus vaccines (OPV) 1 | 50, 751 | 58, 498 | 93,082 | 101,469 | 0.999(0.994-1.004) | 0.764 | 1.004(0997-1.011) | 0.311 | 0.95(0.66-1.24) | 0.543 |
| Oral poliovirus vaccines (OPV) 2 | 52, 456 | 57, 066 | 96,225 | 98,979 | 0.997(0.995-1.000) | 0.017 | 1.007(1.004-1.011) | 0.0001 | 0.76(0.65-0.90) | 0.001 |
| Oral poliovirus vaccines (OPV) 3 | 50, 215 | 55, 012 | 92,110 | 95,423 | 0.997(0.994-1.000) | 0.027 | 1.007(1.002-1011) | 0.001 | 0.79(0.66-0.95) | 0.011 |
| Fully immunized | 51, 477 | 55, 472 | 94,361 | 96, 292 | 1.001(0.996-1.005) | 0.811 | 1.997(0.991-1.005) | 0.500 | 1.13(0.83-104) | 0.432 |

**S2 Table: Segmented regression results showing Relative Risk (RR) for COVID-19, pre-intervention and post-intervention**
