# Supplementary material for: Evaluating the impact of COVID-19 on routine childhood immunizations coverage in Zambia
Source: PLOS Glob Public Health. 2024 Jul 30;4(7):e0003407. doi: 10.1371/journal.pgph.0003407 (PMC11288450; doi:10.1371/journal.pgph.0003407)
Supplement: Attachment — Submitted filename: Response to Reviewers.docx [file pgph.0003407.s010.docx]

**Kelvin Mwangilwa**

**Zambia National Public Health Institute**

**Corner of Chaholi Road Rhodes Park, Lusaka Zambia**

**Dear Marianne Clemence
Staff Editor
PLOS Global Public Health**

We thank the reviewers for their generous comments on the manuscript and have edited, re-analyze the data of manuscript to address their concern.

In particular all of the R scripts and data we wrote is available and I have included multiple links throughout the paper to the appropriate repository.

We believe that the manuscript is now suitable for publications in your Journal.

**Journal Requirements:**

1. We noticed that you used "unpublished data" in the manuscript. We do not allow these references, as the PLOS data access policy requires that all data be either published with the manuscript or made available in a publicly accessible database. Please amend the supplementary material to include the referenced data or remove the references.

The data has been referenced properly see page 18.

2. In the online submission form, you indicated that "The corresponding author can be reached for a reasonable request for the datasets utilized in the current study". All PLOS journals now require all data underlying the findings described in their manuscript to be freely available to other researchers, either 1. In a public repository, 2. Within the manuscript itself, or 3. Uploaded as supplementary information.
This policy applies to all data except where public deposition would breach compliance with the protocol approved by your research ethics board. If your data cannot be made publicly available for ethical or legal reasons (e.g., public availability would compromise patient privacy), please explain your reasons by return email and your exemption request will be escalated to the editor for approval. Your exemption request will be handled independently and will not hold up the peer review process, but will need to be resolved should your manuscript be accepted for publication. One of the Editorial team will then be in touch if there are any issues.

This is well noted. The R script and data set will be available as supplementary materials.

**Major comments**

1. The authors use DHIS2 data to determine immunization coverage as mentioned on page 3, under “data sources”. It would be helpful for the authors to provide a bit more detail about how these data were used. For instance:

1a. What population estimates / denominators were used to estimate coverage from the number of doses delivered, and did those change from month to month / year to year?

This is well noted and has been included. We used static population estimates projected by provided by ZamStats in the 2010 census population.

1b. Were there any issues with incomplete reporting from health facilities, and/or did the completeness of reporting change during any part of the pandemic? Some analysis or discussion of the completeness would be helpful, as there were concerns (especially early in the pandemic) that diversion of workforce to COVID-related activities may compromise data quality and completeness.

This is well noted and a table of the data completeness has been provided. The acceptable report completeness for vaccination is an average of 80% report completeness rate. The data shows that we had a mean value of 94.0% completeness report rate.

2. The analysis plan (page 3) could be of interest to other groups or countries who plan to undertake similar analyses. I have a few questions:

2a. To facilitate further evaluation of the methods, could the authors include the equations that were used in this analysis (i.e. for the regression model) including how they constructed the seasonal/harmonic terms?

The equation has been added and the associated explanation see page 4.

2b. Will the authors plan to deposit the code in a public repository? It could be quite useful for others to use as a template for their own analyses.

This is well noted and am we are happy to share the R script and a data set to help others who may want to use similar methods.

3. The description of the methods on page 3 suggests that the methods estimate a change in slope due to COVID-19, but I’m not sure that I see that happening in the models shown in the various figures. I see that in Table 1 the authors list “trend change”, which I am assuming is the change in the slope. But it seems very odd to me that all the trend changes are essentially 1, even in cases – like rotavirus – where different slopes would seem to allow the model to fit much better than it currently does. If the authors’ current model does theoretically allow the slope to vary, do the authors have any explanation for why all of the slopes are essentially 1?

This is well noted. The data was analyzed to check for this clarification.

For instance, Figure 3 shows the model fit to Rota1 and Rota2 coverage. If the model were really fitting a different slope for each time period, I would expect the post-pandemic period to have a much more negative slope than the pre-pandemic period.

This is well noted and the changes have been made.

Instead, the inclusion of the stockout months in 2022 results in a negative slope that seems to be basically the same for both time periods. In the pre-pandemic months, this slope overestimates the trend in the decline in Rota C, and then underestimates the trend in decline in the post-pandemic period. This also means that the model artificially shows an increase in coverage at the start of the pandemic, when in reality the coverage was about the same, and the modeled “increase” in coverage between March / April 2020 is just an artifact of an overly negative slope in the pre-pandemic time period.

What the reviewer suggests is very important and the team has considered doing this.

If the authors haven’t tried this already, I would suggest that they allow the slope to vary between the two periods, as I think that this may improve the model fit (Rota being an obvious example, but also potentially for other vaccines. If the model is already trying to do this, then it would be good to understand why all the “trend change” is essentially always 1 (same slope before and during pandemic), and with such little uncertainty around that estimated parameter. Perhaps something has gone wrong in the model specification or the model is not converging?

Last, if the authors think that Rota stockouts were not related to the COVID pandemic, then they might also consider excluding these months from the analysis. If they think that the COVID pandemic played a role in stockouts, e.g. via global supply chain issues – then it might be appropriate to retain these months in the analysis. Regardless, however, the fact that the model is not capturing the clear difference in slopes between the pre- and post-pandemic periods needs further exploration and/or explanation.

This has been considered.

4. In the discussion (page 15) the authors write “Indeed our findings are consistent with reports from elsewhere that countries generally reported major disruptions to more than 50% of health services, including services for routine vaccinations [9][30][31].” The authors also write in the conclusion (page 16): “Although immunization programs were still used, there was a large countrywide decline in immunizations during the pandemic.” In general, however, the authors actually find no major effects of the pandemic for most vaccines, aside from MCV2 – or are the authors also considering the Rotavirus stockout to be part of the pandemic? In either case, the authors may want to be more specific about which vaccines were affected and which were not in these sections of the manuscript.

This is explained in page 17. The stock was due to the pandemic as they were disruption in most countries as reported by WHO.

5. In the discussion (page 15), the authors write about the disruptions shown to MCV2 immunization, which are dramatic and persistent. There is some discussion about how this disruption is consistent with other data on first-dose measles disruptions elsewhere. In this analysis, however, the authors show major disruptions to MCV2 but not MCV1. My major question is: why was MCV2 more greatly disrupted than MCV1 – do the authors have any hypotheses that they could share? Is this related to any specific effects of the pandemic on the delivery of vaccines to older children? Were there any changes to policy in Zambia that could have adversely affected the delivery of MCV2 but not MCV1? (The coverage of infant vaccines seems to be otherwise relatively well preserved, aside from rotavirus stockouts).

Our thoughts are that measles dose two coverage is usually lower than dose one because it is administered much later (after 18 months toddler age), this drop within dose two comparison is most likely and certainly made worse for dose two during the pandemic as a result of fear of COVID-19 exposure among mothers and its utilization. See page 17

6. More broadly, the authors find primarily that, for most vaccines, there were no significant changes in coverage due to the pandemic – but there were longer-term declines in coverage that preceded the pandemic and persisted throughout the pandemic. These findings are not explored in much detail in the discussion, and it would be helpful to have some interpretation of these findings.

6a. What do the authors think is driving the longer-term declines in coverage in Zambia, which are certainly troubling?

This is well noted. The Outreach services are a major driver of the vaccine coverage’s in Zambia. A disruption to these services could likely have had a major impact on the coverage. The reasons for the disruption include realignment of both financial and human resources to fighting the pandemic, and refocusing of partner priorities to the pandemic and pandemic preparedness. See page 8

6b. Do the authors have any insight into the strategies that were used to maintain immunization services during the pandemic in Zambia? The ability of the country to maintain immunization delivery for many vaccines during the pandemic is notable from these results. Knowing how the country achieved this would be very valuable!

Policies around leaving a minimum number of staff at health facilities to continue essential services was key to maintaining some gains in the immunization space. See page 18

Minor comments:

1. Have the authors seen the recently published analysis by Winter et al examining the early impact of the COVID-19 pandemic on immunization coverage in Zambia, also published in PLOS GPH? (<https://doi.org/10.1371/journal.pgph.0000554>, Winter AK, Takahashi S, Carcelen AC, Hayford K, Mutale W, Mwansa FD, Sinyange N, Ngula D, Moss WJ, Mutembo S. An evaluation of the early impact of the COVID-19 pandemic on Zambia's routine immunization program. PLOS Glob Public Health. 2023 May 2;3(5):e0000554). This may have been published after the authors prepared their manuscript, and I think that the objectives and time scale covered are different enough that the author’s manuscript is still sufficiently novel. (The other analysis looked only at 2020). Still, it may be worth referencing this manuscript and comparing the two studies’ findings for 2020.

Thank you for this peace of excellent information and it has been well utilized to back up our findings.

2. In the abstract, I wonder if some of the text is missing from the following sentences: “A rise in Rotavirus dose one (RR,1.29; 95% CI: 1.02-1.63, P<. 0.030) with a slop of (RR, 0.98; 95% CI: 0.97-0.99, P<. 0.001), Rotavirus dose two (RR, 1.30; 95% CI: 1.13-1.49, P<. 0.0002) with a slop of (RR, 0.98; 95% CI: 0.98-0.99, P<0.0001) and BCG (RR, 1.00; 95% CI: 0.99- 1.00, P<.0.001).”

This is well noted and the changes have been made.

2a. Should “slop” be “slope”?

It is a slope we are refereeing to.

2b. What do the relative risks mean here? Some appear to relate to the intercepts, and some to the slopes. I would suggest being consistent in reporting either slope, intercept, or both. Also, relative risk is a little bit of a challenging term to use here. I might suggest phrasing these more consistently in terms of “intercepts” (which reflect whether or not there appears to have been an acute disruption at the onset of the pandemic) and “slopes” or “trends” (which reflect whether coverage was increasing or decreasing at different rates in the pandemic period vs the pre-pandemic period).

The confusion have been addressed in the Abstract and in the result section. The data was re-analyzed as advised and the advised where put into consideration. Indeed we relate to the growing or decrease in the slope (trend) in relation to vaccine coverage and the level change due to the impact of covid-19.

2c. I think part of the last sentence may be missing, after Rotavirus dose 2 (what is the vaccine after rotavirus and before BCG?

This is well noted and the changes have been made see page the abstract.

2d. The authors should distinguish between those for which significant increases were seen and those for which coverage stayed the same, i.e. RR close to 1 with 95% CI overlapping 1)

This is well and well reported. See the abstract

3. Page 2: “Concerns about an increase in morbidity and mortality for illnesses other than COVID-19, particularly for childcare services, are being raised as a result of these pauses in the use of health services”. Do the authors mean childcare, or child health?

Thank you for the corrections and changes have been made. The authors meant Child health.

4. Could the authors add line numbers to any revisions of the manuscript, in order to help the reviewers more precisely identify the sections of text of interest?

5. A handful of small grammatical notes:

5a. Page 2, “how people utilize immunization vaccine” – should just be either “immunizations” or “vaccines”

This is well noted and corrections have been made see page 2.

5b. Page 2, “This study sought to determine the effects of COVID-19 on routine immunization utilization as essential health services in Zambia.” – should this be “routine immunization utilization as an essential health service in Zambia”?

This study sought to determine the effects of COVID-19 on routine immunization utilization of as essential health services in Zambia.”

The changes have been made. Reads “This study sought to determine the effects of COVID-19 on routine childhood immunizations coverage in Zambia”. See page 2.

5c. Page 3, “as it was collected at monthly intervals and is consistent with interrupted time series designs[15],[15]” – the ref [15] is duplicated.

Well noted and corrected.

5d. Page 4: “suttle” should be “subtle”

This was well noted and corrected.

Reviewer #2: Similar studies have been conducted in other contexts, however the authors have made a commndable attempt to answer the question using interrupted time series design. I have following suggestions, before it can be recommended for publication.
1.The authors have misspelled slope as "slop" in the abstract. Similalry, the authors misspelled subtle as "suttle" in page 4 of results section. In the third paragrpah of Discussion advent has been mispelled as "advert". The authors need to copy-edit the whole manuscript for readability.

This has been corrected see page 17

2. The authors should define outcome measure "coverage" in the methods section. What were the numerator and denominator for calculating coverage. You may use this paper for reference. "MacDonald SE, Russell ML, Liu XC, Simmonds KA, Lorenzetti DL, Sharpe H, Svenson J, Svenson LW. Are we speaking the same language? An argument for the consistent use of terminology and definitions for childhood vaccination indicators. Human vaccines & immunotherapeutics. 2019 Mar 4;15(3):740-7."

This is well noted and this has been clarified. Further, we used static population estimates provided by the Zambia Statistics Agency (ZamStats ) from the 2010 projected census population estimates to account for the denominators and estimate coverage, which changed from one year to another year due to population growth with each month having the same denominator by dividing the projected population for each year by twelve months

How was the yearly cohort of children for calculating coverage was determined? was it based on the month/year of birth, please refer to this paper: MacDonald SE, Paudel YR, Kiely M, Rafferty E, Sadarangani M, Robinson JL, Driedger SM, Svenson LW. Impact of the COVID-19 pandemic on vaccine coverage for early childhood vaccines in Alberta, Canada: a population-based retrospective cohort study. BMJ open. 2022 Jan 1;12(1):e055968.

The population is based on the yearly population divided by 12 months, as the projections population are not assumed to change from one month to the other but on yearly basis. See page 3.

3. Similarly, when was the number of doses of vaccine administered determined. For vaccines that have multiple doses i.e DTAP, how was 1st, second and third dose determined? need to be explained.

Our data is routinely administered and therefore we depended on what was given by DHIS2. Each dose is captured every month and reported in the system in most case by the end of each month.

4. How was mean monthly count, monthly rates were calculated need to be explained in the methods section.

In order to calculate the mean monthly rate we captured trends in eligible populations and corresponding coverage, we included monthly vaccination patterns in 2017 through to 2022

5. The authors use a term "step change" in Table 1, is it the same as "slope change" or different concept. Need to maintain consistency throughout the manuscript.

This is well noted step change is the level change while trend change is the slope change

6. The authors use a figure title" Changes in Pentavalent dose 1, 2 & 3 following the intervention of COVID-19 pandemic" however they report actual coverage figure they calculated before and after the pandemic. The figure titles need to be revised as " xxx vaccine coverage before and after COVID-19 pandemic" or something like that.

This is well noted and the changes have been made see all the figures.

7. The authors need to present the R scripts with clear instructions used for the analysis for transparency and replicability of the study. The link for secondary data from web sources also may be publicly shared.

This is well noted and the R script will be share with clear instructions.

8. The authors use a term "risk ratio; and "rate ratio" interchangeably, please use consistent term and define the term in methods section what it means? and how was thie determined.

This has been clarified is relative risk that we are using. This was determined by Poisson regression model. See the method section.

9. The authors make an analogy for COVID-19 as a natural intervention. However, the authors may not need to repeat the term "COVID-19 intervention" Mutiple times.

This is well noted and changes have been made. See page 8-16.

10. What could be the reason for no change in BCG, OPV and pentavalent coverage. If the measure used is not reflecting the vaccine uptake this needs to be mentioned in the limitations.

Policies around leaving a minimum number of staff at health facilities to continue essential services was key to maintaining some gains in the immunization space. See page 23

**Mr Kelvin Mwangilwa**

**Lead Statistician**

**Zambia National Public Health Institute**

**On behalf of all authors**
