# Supplementary material for: Evaluating the impact of COVID-19 on routine childhood immunizations coverage in Zambia
Source: PLOS Glob Public Health. 2024 Jul 30;4(7):e0003407. doi: 10.1371/journal.pgph.0003407 (PMC11288450; doi:10.1371/journal.pgph.0003407)
Supplement: Attachment — Submitted filename: Response_to_Reviewers.docx [file pgph.0003407.s011.docx]

**Kelvin Mwangilwa**

**Zambia National Public Health Institute**

**Corner of Chaholi Road Rhodes Park, Lusaka Zambia**

**Dear Nyamongo Onkoba, Ph.D
Academic Editor
PLOS Global Public Health**

We thank the reviewers for their generous comments on the manuscript and we have edited to address the concerns raised.

2. Regarding the analysis plan: Thanks to the authors for adding the description of the harmonic terms (that two sin and cosine pairs were added), and for the addition of the equations used in the analysis. This description is very helpful. One additional clarification: did the authors use a two-step process, where they first adjusted for seasonality using the harmonic terms, and then used the seasonally-adjusted T as the input into the ITS model? Or did the ITS model itself contain the harmonic terms? Either approach could be reasonable, but I would suggest that the authors clarify.

This is well noted and indeed good guidance. It was a two-step process and we have included as guided.

My sense from the description is that a two-step process was used, and in that case, it would be sufficient just to add a brief statement explaining that this was a two-step process (and that T in the ITS analysis is seasonally adjusted – perhaps adding a note to this effect under point (i) in the equation description). But if the seasonal terms were included in the ITS regression itself (e.g. if the equation for the ITS regression was truly Yt = b0 + b1T + b2sin(2pi/12*T) + b3cos(2pi/12*T)… ), then it would be useful to re-write the ITS equation to fully show these sin/cosine harmonic terms as well.

The guidance is well received and the changes have been made. We have re-written the equation as guided.

3. Previous major comment #3, regarding the interpretation of changes in slope / trends. Thanks to the authors for clarifying in the text. The addition of the ITS equations also is helpful in clarifying how to interpret these results. A few additional comments:

a. Note that the previous Table 1 (Segmented regression results…) should now be re-labeled as Table 2 as a new Table 1 has been added.

Thank you and the changes have been made.

b. The “Segmented regression results” table is cut off in the PDF version of the file that I have access to, unfortunately – can it be resized so that we can see the additional columns? In particular, I am not sure what the additional RR column that is cut of represents – does this show whether the slopes in the pre- and during-COVID periods are significantly different? Or does it show whether the level is different (i.e. the intercept b2?) Ideally I think that this table would show all of these – i.e. (1) the trends (slopes) both pre-COVID and during COVID, (2) whether the slopes are different before and after COVID, and (3) whether the levels (intercept) are different before and after COVID. Perhaps it already does, but I can’t see it because the table was cut off in the PDF conversion?

We apologies for this on the PDF. A new PDF has been provided. Indeed the table show the trends (slopes) both pre-COVID and during COVID, whether the slopes are different before and after COVID, and whether the levels (intercept) are different before and after COVID.

c. The RR (95% CI) header for the slope change pre-covid-19 column in the “segmented regression results” table doesn’t have a superscript (c), indicating that the effects are adjusted for seasonality, but the RR column for the slope change during COVID-19 does have that superscript. Should both have the superscript if both are adjusted for seasonality?

This is well noted and the superscript (c) is indicated.

4. Previous major comment #4, regarding the discussion and clarifying that measles (2nd dose) and rotavirus were those affected. I appreciate the authors’ clarifications in the discussion, no further comments.

5. Previous major comment #4, regarding the specific disruptions to MCV2 coverage. Thanks to the authors for their additions to the manuscript. The authors propose that these drops may be due to fear of COVID-19 among mothers and decreased health service utilization. The manuscript doesn’t quite specify, however, why this would have affected MCV2 more than MCV1. Could the authors add a sentence explaining why they think that these factors affected MCV2 more than MCV1? (for instance, is this because parents might perceive that the MCV2 dose is less important to protect their child than the MCV1 dose, and therefore the risk/benefit calculations in comparison to COVID exposure may have meant that they were more likely to delay or skip MCV2 than MCV1? Or are there other mechanisms?)

The good advice is well noted and clarifications have been made on the questions raised. The following was added. This could be that parents might perceive that the Measles dose two is less important to protect their child than the Measles dose one, and therefore the risk benefit in comparison to COVID-19 exposure may have meant that they were more likely to delay or skip Measles dose two than Measles dose one. Parents who, having raised multiple children over the years, have grown accustomed to their child's healthcare provider and may now downplay the significance of vaccinations. See page 11.

Minor comments:

Previous minor comment #2, regarding the abstract. Thanks to the authors for the changes, which I think are very helpful. I note that the third sentence of the results still seems to have some words that are missing. Should this read “A decreased slope **was observed** in Rotavirus dose one (RR, 0.966; 95% CI: 0.957-0.976) and Rotavirus dose two (RR, 0.966; 95% CI: 0.950-0.983).”?

This was well noted and thank you. The sentence has been re-written. Another drop in immunization coverage was observed in Rotavirus dose one (RR, 0.966; 95% CI: 0.957-0.976) and Rotavirus dose two (RR, 0.966; 95% CI: 0.950-0.983). See the abstract.

Previous minor comment #3: Thanks to the authors for their response. I still see “childcare services” in the Introduction, in the sentence “Concerns about an increase in morbidity and mortality for illnesses other than COVID-19, particularly for childcare services, are being raised as a result of these pauses in the use of health services”. Would suggest that this be changed to “child health”.

This is well noted and the changes were was made it is now written child health. See page 2.

**Mr Kelvin Mwangilwa**

**Lead Statistician**

**Zambia National Public Health Institute**

**On behalf of all authors**
