# Supplementary material for: Evaluating the impact of COVID-19 on routine childhood immunizations coverage in Zambia
Source: PLOS Glob Public Health. 2024 Jul 30;4(7):e0003407. doi: 10.1371/journal.pgph.0003407 (PMC11288450; doi:10.1371/journal.pgph.0003407)
Supplement: Attachment — Submitted filename: Plos Global Health Final comments.docx [file pgph.0003407.s012.docx]

Reviewer #1: Thanks to the authors for their revisions. I have two remaining clarifications, again regarding the harmonic / seasonal terms in the ITS analysis.
Question #1:
Originally, the equation was given as:

"Yt =b0 + b1T + b2Xt + b3TXt, Where b0 represents the baseline level at T = 0, b1 is interpreted as the change in outcome associated with a time unit increase (representing the underlying pre-COVD-19 trend), b2 is the level change following the COVID-19 period and b3 indicates the slope change following the COVID-19 (using the interaction between time and COVID-19: TXt)"

I had initially asked about how exactly the seasonality adjustment was performed - was it a two-step process, whereby the harmonic sin/cos pairs were used to generate a seasonally adjusted value for T and then the regression ("Yt =b0 + b1T + b2Xt + b3TXt") was fit? Or were the harmonic terms included directly in the regression equation in a single step?

The authors write that they used a two-step process, but then write the equation as it were a single large regression that was fit in a single step process (following the example that I had written - sorry if the example equation caused confusion!).

The manuscript now reads:
"In standard ITS analyses, the following segmented regression model is used: Yt = b0 + b1T + b2sin(2pi/12*T) + b3cos(2pi/12*T), Where b0 represents the baseline level at T = 0, b1 is interpreted as the change in outcome associated with a time unit increase (representing the underlying pre-COVD-19 trend), b2 is the level change following the COVID-19 period and b3 indicates the slope change following the COVID-19 (using the interaction between time and COVID-19: TXt) [22]."

This isn't correct, however, as the interpretation of the covariates b1, b2, b3, etc. would all change with the revised equation, and no longer would have the same meaning as in the original equation.

From the code provided (line 190), I now see that there was indeed a one-step adjustment used; that is, that the regression equation directly included the harmonic terms. The code is written as:

model3 <- glm(bcg ~ offset(log(stdpop)) + covid19 + time + harmonic(month,2,12),
family=quasipoisson, data)

where harmonic is (I think) from the tsModel package. From that documentation, harmonic(month,2,12) creates two *pairs* of sin/cos terms - is that right? The source code suggests that this is done by generating sin(x*n*(2pi/period)), where x is your variable of interest (in this case, month), and n = 1, 2, 3 ... N (up to the number N of pairs of sin/cos terms that are added). See <https://rdrr.io/cran/tsModel/src/R/tsModelSpec.R#sym-harmonic> for the underlying code.

If that's true - and I would ask the authors to check this logic closely - then I think that the equation should look something like:
Yt =b0 + b1T + b2Xt + b3TXt + b4sin((2pi/12)*m) + b5cos((2pi/12)*m) + b6sin((4pi/12)*m) + b7cos((4pi/12)*m)

Where b0 represents the baseline level at T = 0, b1 is interpreted as the change in outcome associated with a time unit increase (representing the underlying pre-COVD-19 trend), b2 is the level change following the COVID-19 period, b3 indicates the slope change following the COVID-19 (using the interaction between time and COVID-19: TXt), m is the calendar month, and b4-b7 are coefficients on the seasonal adjustment terms.

Is that right? Sorry again for the confusion, and please check this logic closely to make sure it matches the actual analysis that was conducted. I think that it is important to make sure that the equation is accurately written so that the analysis can be interpreted correctly.

This is true and very logical. Great appreciations to the reviewer for the very important guidance. We have taken time to re-analysis our data with the interaction term in the equation just as the reviewer recommended which is the main idea of this analysis and thank you for taking time to educate the team. The revised R script make a lot of sense and we used it to re-analyze the work. Thank you for positive feedback. We have used the following which speaks to this analysis and we do agree with what the reviewer suggest and we have further changes our estimates and figure that speak analysis.

Yt =b0 + b1T + b2Xt + b3TXt + b4sin((2pi/12)*m) + b5cos((2pi/12)*m) + b6sin((4pi/12)*m) + b7cos((4pi/12)*m)

Where b0 represents the baseline level at T = 0, b1 is interpreted as the change in outcome associated with a time unit increase (representing the underlying pre-COVD-19 trend), b2 is the level change following the COVID-19 period, b3 indicates the slope change following the COVID-19 (using the interaction between time and COVID-19: TXt), m is the calendar month, and b4-b7 are coefficients on the seasonal adjustment terms.

Question #2:
This does bring up one other question: the authors initially wrote that their equation included an interaction term between COVID and time:

"Yt =b0 + b1T + b2Xt + b3TXt, Where b0 represents the baseline level at T = 0, b1 is interpreted as the change in outcome associated with a time unit increase (representing the underlying pre-COVD-19 trend), b2 is the level change following the COVID-19 period and b3 indicates the slope change following the COVID-19 (using the interaction between time and COVID-19: TXt)"

But, as above, the code in line 190 doesn't include this interaction term:
model3 <- glm(bcg ~ offset(log(stdpop)) + covid19 + time + harmonic(month,2,12),
family=quasipoisson, data)

Am I looking at the wrong line in the code, or is there another reason for the discrepancy? Based on the equation in the text, I would have expected to see:

model3 <- glm(bcg ~ offset(log(stdpop)) + covid19 + time + time*covid + harmonic(month,2,12),
family=quasipoisson, data)

I have provided a simplified version of the authors’ code, which just runs model 3 for Rota dose 1 (without the interaction term). This produces results that look quite similar to the authors’ figures (see attached model3.png, which is quite similar to the authors’ Figure 3).

However, when I run a new model (model 4 in the attached code), but include the interaction terms, I see a very different result (see model4.png). As expected, this allows the slope to vary between the COVID and post-COVID periods, with very different interpretations of the ITS analysis.

Can the authors clarify – was the interaction term inadvertently excluded from the analysis here? If so, it seems like the interaction term would need to be included in order to be able to interpret any of the “trend change” results in the manuscript.

Apologies that it took me several revisions to raise this query, as it required me to dive into the code to understand the seasonality terms before I noticed the discrepancy.

We appreciate the great feedback from the reviewer your guidance is very clear and the team share your great thoughts. The code you have provided adds the interactions term which we do agree with and have considered the final model below. All the results are coming from the model provided below as advised in the R script which makes a lot of logic. Indeed the final model includes time interaction and thank you for this educative thought and a simplified R script.

model4 <- glm(Rota1 ~ offset(log(stdpop)) + covid19 + time + covid19*time + harmonic(month,2,12),

family=quasipoisson, data)
