# Supplementary material for: Evaluating the impact of COVID-19 on routine childhood immunizations coverage in Zambia
Source: PLOS Glob Public Health. 2024 Jul 30;4(7):e0003407. doi: 10.1371/journal.pgph.0003407 (PMC11288450; doi:10.1371/journal.pgph.0003407)
Supplement: Attachment — Submitted filename: Reviewer report on Childhood immunization and COVID-19.docx [file pgph.0003407.s013.docx]

Dear Editor-in- Chief,

I have provided my comments on the manuscript, PGPH-D-23-00790R3 titled, “**Evaluating the impact of COVID-19 on routine childhood immunization coverage in Zambia**”

The manuscript presents described impact of COVID-19 on routine childhood immunization coverage in Zambia. The manuscript is generally well written and presented.

Other general and specific observations follow:

1. Originality of value: The manuscript presents an original study that was designed and conducted Zambia. The paper addressed a relevant public health issue, namely childhood immunization and COVID-19 with thought-provoking results.

2. Suitability and soundness of technique: The techniques used for the study are adequate and robust. It utilized longitudinal design using the national routine immunization data. Interrupted time series analysis was performed to quantify changes in immunization utilization with adjusted segmented Poisson regression model

3. Clarity of Presentation: The manuscript was very clear in narrative presentation. It is easy to follow and complied with the journal specifications.

4. Areas requiring correction: While the paper is generally well written, some corrections are required, which have been highlighted for the authors' attention below.

**Recommendation:** The paper should be accepted after minor revision

Thank you.

Yours sincerely,

**Olaoye Titilayo PhD**

Abstract

Under the method: This statement should be change “we used seasonally adjusted segmented Poisson regression model” to seasonally adjusted segmented Poisson regression model was utilized.

Results:

Result for the other routine immunization should be include in the abstract

Conclusion: It seem not to tally with the findings based on this statement in the result “Utilization of health services was similar with historical levels prior to the first case of COVID-19”

The authors should provide specific recommendation that can be used to address the issues not just stating that “proactive and focused immunization strategies are required to counteract these impacts”

Introduction:

The authors should include information on how the routine immunization coverage was like before the COVID-19 in term of the uptake. The rotavirus vaccine shortage should also be included in the introduction.

Method:

The authors should clarify is the interrupted time series is the analysis performed or the study design. What is in the method section is different from the abstract.
