# Supplementary material for: Evaluating the impact of COVID-19 on routine childhood immunizations coverage in Zambia
Source: PLOS Glob Public Health. 2024 Jul 30;4(7):e0003407. doi: 10.1371/journal.pgph.0003407 (PMC11288450; doi:10.1371/journal.pgph.0003407)
Supplement: Attachment — Submitted filename: Responce to Reviewers_Final.docx [file pgph.0003407.s014.docx]

**Dear Editor-in- Chief,**

**Reviewer 1 Comments to the Author**

1. Did the authors revise all of the results in the manuscript with the new regression results? For instance, the first paragraph of the results states that “Monthly Measles 2 vaccinations was 0.67 times lower during the COVID-19 pandemic (95%CI=0.61-0.74) and declined by 0.998 per month (95% CI=0.997-1.005). However, period between March 2020 and December 2022, Measles 2 vaccines reduced significantly by about 0.2% (RR=0.998, 95% CI: 0.997-1.005).” This is the same as in the previous submission and I am not sure how it relates to the new Table 2, which states that the slope change pre-COVID-19 was 0.998 (0.995-1.003), the slope change during COVID-19 was 1.00 (0.997-1.010), and the step change was 0.59 (0.43 – 0.80). I wonder if the authors have not updated this from the previous analysis with the old, incorrect regression? The discussion of MCV2 in the discussion section appears to have the updated results included, so this may have been just an omission. (Also, the second sentence seems to repeat results in the first sentence, and this could probably be consolidated).

This is well noted and thank you for the advice the changes have been made, it now reads “Monthly Measles 2 vaccinations slope change pre-COVID-19 was 0.998 (0.995-1.003), the slope change during COVID-19 was 1.00 (0.997-1.010), and with a significant step change of about 41% (RR=0.59, 95%CI: 0.43-0.80)”.

Also, in Table 2, most of the rows appear to have been updated with the new regression results. I believe, however, that the last row (Fully Immunized) has not changed between the previous version of the manuscript and this one. Was this to be expected (e.g. did the fully immunized analysis run for the previous manuscript version already include the appropriate interaction terms)? I mention this just to make sure that it is not inadvertent, e.g. that this row wasn’t updated like the others as the authors made their revisions.

Thank you for the advice we looked through the results indeed this was omitted when updating the paper and the results indeed look different and here we have made changes after re-analyzing the data.

In general, I would suggest that the authors carefully review all of the results in the manuscript to make sure that they have been updated for the new analysis.

2. The manuscript would benefit from some additional proofreading; defer to the editors on this. For instance, there are two mentions of “slop” which should be “slope” in the results section of the abstract.

This has been rectified and thank you for the advice.

**Reviewer 2 comments to the authors**

Abstract

Under the method: This statement should be change “we used seasonally adjusted segmented

Poisson regression model” to seasonally adjusted segmented Poisson regression model was

utilized.

Thank you and the changes have been made.

Results:

Result for the other routine immunization should be include in the abstract

Thank you and the details were added.

Conclusion: It seem not to tally with the findings based on this statement in the result

“Utilization of health services was similar with historical levels prior to the first case of COVID-

19”

The authors should provide specific recommendation that can be used to address the issues not

just stating that “proactive and focused immunization strategies are required to counteract these

impacts”

Thank you for the advice and the changes have been made.

Introduction:

The authors should include information on how the routine immunization coverage was like

before the COVID-19 in term of the uptake.

This well noted and the information has been included.

The rotavirus vaccine shortage should also be included in the introduction.

This is well noted and the literature has been included.

Method:

The authors should clarify is the interrupted time series is the analysis performed or the study

design. What is in the method section is different from the abstract.

Thank you for the advice and the changes have been made and clarified in the method section.

Yours sincerely on behalf of other authors

Kelvin Mwangilwa
